# Neurological Complications after Lateral Transpsoas Approach to Anterior Interbody Fusion with a Novel Flat-Blade Spine-Fixed Retractor

**DOI:** 10.1155/2016/8450712

**Published:** 2016-05-12

**Authors:** Pierce Nunley, Faheem Sandhu, Kelly Frank, Marcus Stone

**Affiliations:** ^1^Spine Institute of Louisiana, Shreveport, LA 71101, USA; ^2^Medstar Georgetown University Hospital, Washington, DC 20007, USA

## Abstract

*Introduction*. The lateral lumbar interbody fusion (LLIF) surgical approach has potential advantages over other approaches but is associated with some unique neurologic risks due to the proximity of the lumbosacral plexus. The present study analyzed complications following LLIF surgical approach using a novel single flat-blade retractor system.* Methods*. A retrospective data collection of patients receiving LLIF using a novel single flat-blade retractor system at two institutions in the US. Inclusion criteria were all patients receiving an LLIF procedure with the RAVINE® Lateral Access System (K2M, Inc., Leesburg, VA, USA). There was no restriction on preoperative diagnosis or number of levels treated. Approach-related neurologic complications were collected and analyzed postoperatively through a minimum of one year.* Results*. Analysis included 253 patients with one to four treated lateral levels. Immediate postoperative neurologic complications were present in 11.1% (28/253) of patients. At one-year follow-up the approach-related neurologic complications resolved in all except 5 patients (2.0%).* Conclusion*. We observed an 11.1% neurologic complication rate in LLIF procedures. There was resolution of symptoms for most patients by 12-month follow-up, with only 2% of patients with residual symptoms. This supports the hypothesis that the vast majority of approach-related neurologic symptoms are transient.

## 1. Introduction

Lumbar spinal fusion has historically been accomplished through open surgical procedures [1].

The first minimally invasive spine (MIS) procedure, chemonucleolysis, needle injected chemical chymopapain into the annulus of a herniated disc was in 1969 [2]. However, the first MIS fusion procedure was not until the introduction of the MIS stand-alone anterior lumbar interbody fusion (ALIF) in 1995 [1, 3]. ALIF with posterior fixation has become an accepted method of stabilizing and fusing the spine, but it is associated with significant complications and, in most cases, the need for an access surgeon to expose the spine [4–6].

The MIS lateral transpsoas approach of lateral lumbar interbody fusion (LLIF) was introduced in 1998, in an effort to reduce anterior approach-related complications [7]. The LLIF procedure utilizes a lateral, retroperitoneal, transpsoas approach coupled with neuromonitoring [7, 8]. LLIF has unique approach-related complications, such as transient nerve deficits, nerve injury with residual effects, and contralateral motor deficits [8–11]. To date, there is a growing body of literature reporting the complications associated with the LLIF surgical approach. However, there is a paucity of literature that analyzes the specific retractor system utilized for the approach. The present study analyzed complications following LLIF surgical approach using a novel single flat-blade retractor system.

## 2. Materials and Methods

With institutional review board approval, patients were retrospectively identified in chart review from two centers. Identified patients underwent MIS LLIF between October 2010 and August 2014. Inclusion in the study was restricted to patients receiving lateral lumbar interbody fusion using the RAVINE Lateral Access System, K2M, Inc., Leesburg, VA, USA (RAVINE), with minimum of one-year follow-up and complete data to evaluate approach-related neurologic complications at follow-up. Patient preoperative diagnosis was not restricted and included degenerative disc disease with/without spondylolisthesis and deformity cases.

**Figure 1:**
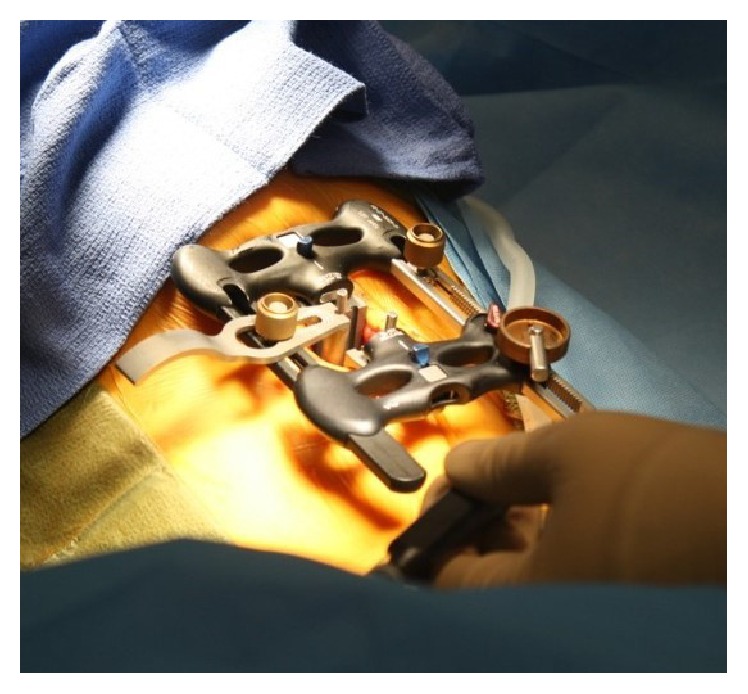
Intraoperative photograph of RAVINE.

**Figure 2 fig2:**
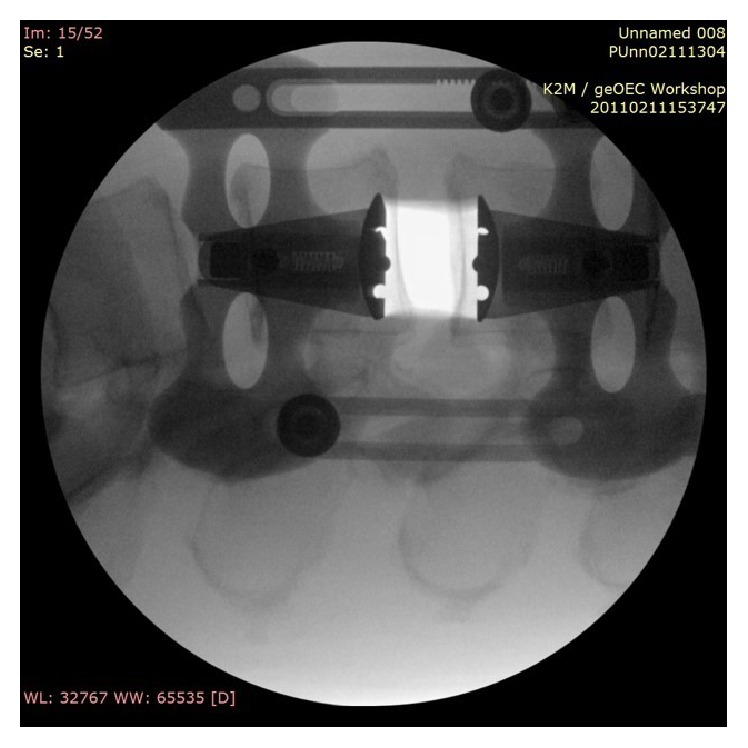
Intraoperative radiograph of RAVINE.

**Figure 3:**
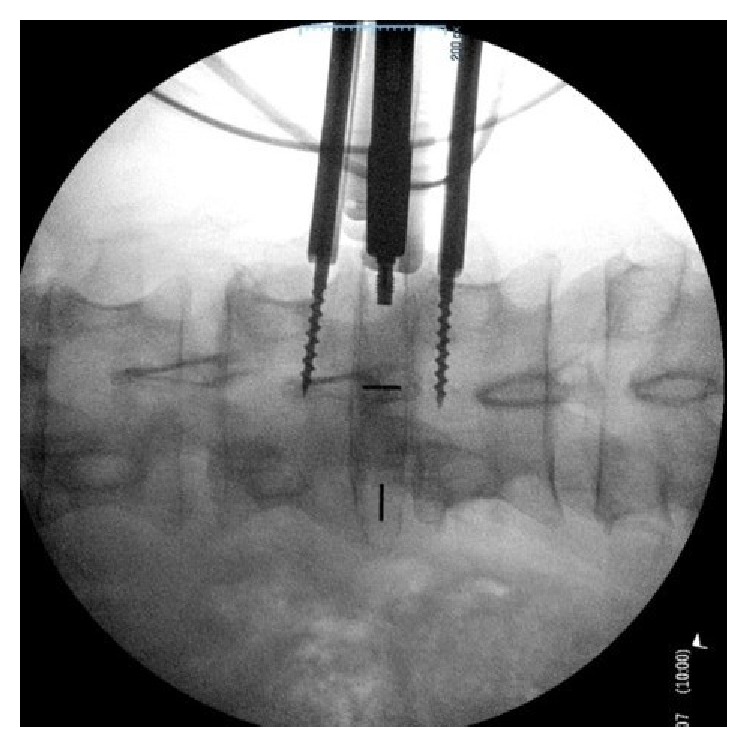
Intraoperative radiograph of PEEK Cage Insertion using RAVINE.

RAVINE is used for a transpsoas approach with rigid fixation to the spine. The system uses dual flat blades with options for a third and fourth blade, instead of the standard tubular retractor. Radiographic intraoperative images and photos are included, Figures 1 [Fig fig2]–3.

The surgeries were performed at two centers in the US between October 2010 and August 2014 by an orthopedic and a neurosurgeon, both fellowship trained spinal surgeons experienced in performing lateral access surgery. The lateral surgery was performed with the surgeon's choice of interbody cage while using RAVINE. The surgical procedure differed based on surgeon preference, and thus surgeries included LLIF with posterior fixation (rods and percutaneous screws) as well as stand-alone LLIF. The LLIFs with posterior fixation surgeries were completed as same-day procedures in some cases and staged over multiple days in other cases. All LLIF surgeries were performed with the guidance of neuromonitoring. The two surgeons in this study included the use of neuromonitoring per their standard protocol for lumbar LLIF cases. This included monitoring L2, L3 (rectus femoris), L3, L4 (vastus medialis), L4 (tibialis anterior), L5, S1 (peroneus longus), and S1, S2 (gastroc to gastrocnemius) for all cases. Baseline measures were obtained and stimulation was provided multiple times during the decompression and insertion of the lateral device. Following surgery, neurologic complications were collected and evaluated for relationship to the surgical approach at each postoperative visit.

Data were retrospectively collected and included demographics, operative details, and postoperative neurologic complications. Descriptive statistics were calculated for the demographic and operative data. All neurologic complications were analyzed for relationship to the surgical approach and resolution at final follow-up. SPSS Statistics 20 was used to run multinomial regression analysis of neurologic complications with surgical time, EBL, and specific levels treated.

## 3. Results

Retrospective data were collected and analyzed for 253 patients meeting the inclusion/exclusion criteria, with an average follow-up of 13.2 months at the final postoperative visit. The average age at time of surgery was 61 years and 31% of patients were male. Complete patient demographics are included in Table 1.

Surgical procedure differed based on surgeon preference including same-day LLIF with posterior fixation, staged LLIF with posterior fixation, and stand-alone LLIF, so operative data were analyzed separately for each surgical procedure. Hospital length of stay was similar between same-day LLIF with posterior fixation and stand-alone LLIF. The posterior portion of the staged LLIF procedure was performed one day after the lateral surgery, with the length of stay five days longer on average than same-day procedure. Surgery time and EBL were also noticeably longer than same-day and stand-alone procedures. Complete operative results are included in Table 2.

The inclusion/exclusion criteria included all patients treated with RAVINE and complete data to evaluate for any approach-related neurological complications; therefore, the lateral and posterior treated levels varied. The most treated lateral and posterior level was L4-L5 followed by L3-L4. All treated lateral and posterior levels are reported in Table 3.

Analysis of neurologic complications showed transient approach-related symptoms existing in 11.1% (28/253) of patients at the initial postoperative visit. Femoral cutaneous nerve neuropraxia was the most common symptom. By the last postoperative visit, neurologic symptoms remained in only 2.0% (5/253) of patients. The symptoms included foot drop, hypersensitivity in the left lateral thigh, left thigh numbness, left thigh tingling, and right thigh dysesthesia. The patient that experienced foot drop was a grade 3 spondylolisthesis prior to surgery and received same-day LLIF with posterior fixation at L4-L5. The patient with hypersensitivity in the left lateral thigh also received same-day LLIF with posterior fixation at L4-L5. Left thigh numbness was experienced after staged two-level LLIF at L3–L5 with posterior fixation from L3–S1. Tingling in the left thigh occurred after staged three-level LLIF at L2–L5 with posterior fixation from L2–S1. The last patient, with chronic right thigh dysesthesia, received a stand-alone three-level LLIF from L2–L5. Review of neuromonitoring of patients with complications did not reveal any relationship between intraoperative alarm and presence of complications. Multinomial regression analysis indicated that there was no statistically significant relationship between neurologic complications and surgery time, EBL, or levels treated.

## 4. Discussion

The LLIF surgical approach has potential advantages when compared to ALIF, TLIF, and PLIF but is associated with some unique risks. The proximity to the lumbosacral plexus increases the risk of neurologic complications, with the literature reporting a wide range of complications 0.7–23% [9, 12–16].

An early retrospective review of the direct lateral transpsoas approach of 58 patients at one-year follow-up showed that two (3.4%) patients experienced persistent motor deficit from L4 nerve injury [8]. Lykissas et al. hypothesized that neurologic deficits following LLIF surgery decrease over time and, in a review of 919 LLIF treated levels at 18 months, approach-related deficits were reduced to 3.2% of patients [17]. A prospective multicenter study of 107 patients also found that postoperative weakness appeared to be transient. Initially, 33.6% of patients had weakness, but symptoms resolved by six months in all patients except 7 (6.5%) [14]. Pumberger et al. found in 235 LLIF patients that motor deficits occurred in 2.9% of patients at 12-month follow-up [12].

A retrospective case series of 118 patients receiving one-level to four-level LLIF reported a postoperative complication rate of 36% that was reduced to 0.8% (one patient) at the final follow-up [18]. Data from an extreme lateral interbody fusion (XLIF) of 107 patients showed lower extremity weakness in 24% (36/107) of patients following surgery. Five (5%) patients had continued motor weakness at 12 months. However, by 24 months 4/5 deficits were only a single motor grade and one patient was lost to follow-up [19]. The current study results follow a similar trend with an immediate postoperative neurologic complication rate of 11.1% (28 patients) reduced to 2.0% (5 patients) at last follow-up.

Recent studies have attempted to isolate the risk factors for neurological deficits following LLIF surgery. Rodgers et al. did not find a correlation between the number of levels and neurologic deficits but did find a statistically significant relationship to surgery at the L4-L5 level [9]. However, other studies and our study did not reproduce this correlation [12]. The complications reported here occurred at L4-L5 for 2 patients, L2–L5 for two patients, and L3–L5 for one patient, but no correlation exists to surgery time, EBL, or levels treated.

A case series of 107 patients found that the number of levels operated was the strongest predictor of complications, with an additional correlation of hip flexor weakness to surgery time [14]. However, our study did not analyze the relationship between the number of levels operated, the specific levels operated, and the surgery time.

This study does have limitations, largely related to its retrospective design. A multicenter prospective study is currently enrolling to further study outcomes and complications following LLIF surgery with RAVINE.

## 5. Conclusion

Minimally invasive spine surgery is associated with risks, regardless of the approach. Although some reports in the literature have found correlations of certain risk factors such as levels treated, number of levels treated, and surgery time, with postoperative neurologic complications, this study data did not support those reports. The LLIF procedure minimizes the risks of anterior approach such as damage to major blood vessels resulting in potentially catastrophic blood loss and retrograde ejaculation. However access through the psoas in LLIF has potential neurologic complications. Recent literature concludes that neurologic complications following LLIF surgery are predominantly transient. The LLIFs performed here, using RAVINE Lateral Access System, add to the body of literature that most neurologic complications are transient and resolve by 12-month follow-up.

## Figures and Tables

**Table 1:** Demographics.

Sex	Male: 78, female: 175
Age (years)	61 (range 28–86)
Height (in)	65.9 (range: 51–76)
Weight (lbs)	181.4 (range: 107–301)
BMI (kg/m^2^)	29.3 (range: 19.0–43.9), not noted: 9
Current smoker	Yes: 47, no: 183, not noted: 23

**Table 2:** Operative data.

Lateral and posterior same-day (*N* = 210)	
LOS (days)	2.36 (range: 1–10)
Surgery time (min)	150.0 ± 56.2 (range: 58.0–360.0)
EBL (cc)	87 (range: 10–700)

Lateral and posterior staged (*N* = 17)	

LOS (days)	7.41 (range: 3–15)
Surgery time (min)	453.2 ± 85.0 (range: 299.0–582.0)
EBL (cc)	262 (range: 50–1350)

Lateral stand-alone (*N* = 26)	

LOS (days)	3.88 (range: 1–13)
Surgery time (min)	122.2 ± 38.9 (range: 77.0–216.0)
EBL (cc)	55.2 (range: 15–300)

**Table 3:** Lateral and posterior levels treated.

	Lateral levels treated	Number of patients, lateral	Number of patients with neurological complication^*∗*^
One	L1-L2	3	0
	L2-L3	19	0
	L3-L4	52	0
	L4-L5	85	2
	L5-S1	1	0

Two	T12–L2	1	0
	L1–L3	4	0
	L2–L4	17	0
	L3–L5	34	1
	L4–S1	3	0

Three	L1–L4	1	0
	L2–L5	25	2
	L3–S1	1	0

Four	L1–L5	1	0
	L2–S1	6	0

^*∗*^At final postoperative visit (average follow-up of 13.2 months).
